# The Phagocytic Function of Macrophage-Enforcing Innate Immunity and Tissue Homeostasis

**DOI:** 10.3390/ijms19010092

**Published:** 2017-12-29

**Authors:** Daisuke Hirayama, Tomoya Iida, Hiroshi Nakase

**Affiliations:** Department of Gastroenterology and Hepatology, Sapporo Medical University School of Medicine, Minami 1-jo Nishi 16-chome, Chuo-ku, Sapporo, Hokkaido 060-8543, Japan; d.hirayama@sapmed.ac.jp (D.H.); tomoya.iida.0306@gmail.com (T.I.)

**Keywords:** macrophages, innate immunity, phagocytosis

## Abstract

Macrophages are effector cells of the innate immune system that phagocytose bacteria and secrete both pro-inflammatory and antimicrobial mediators. In addition, macrophages play an important role in eliminating diseased and damaged cells through their programmed cell death. Generally, macrophages ingest and degrade dead cells, debris, tumor cells, and foreign materials. They promote homeostasis by responding to internal and external changes within the body, not only as phagocytes, but also through trophic, regulatory, and repair functions. Recent studies demonstrated that macrophages differentiate from hematopoietic stem cell-derived monocytes and embryonic yolk sac macrophages. The latter mainly give rise to tissue macrophages. Macrophages exist in all vertebrate tissues and have dual functions in host protection and tissue injury, which are maintained at a fine balance. Tissue macrophages have heterogeneous phenotypes in different tissue environments. In this review, we focused on the phagocytic function of macrophage-enforcing innate immunity and tissue homeostasis for a better understanding of the role of tissue macrophages in several pathological conditions.

## 1. Introduction

Macrophages have a defensive function against pathogens such as microbes, and play an important role in the homeostatic maintenance of the body through the disposal of internal waste materials and tissue repair. However, as macrophages have the ability to present antigens to T cells and function as effectors for cell-mediated immunity, it is known that they affect the development of infectious diseases, cancers, and chronic inflammatory diseases such as arteriosclerosis. Additionally, phagocytosis plays a critical role in that process. Macrophages exist in all vertebrate tissues, and different stimuli will affect macrophage phenotypes differently. Mills et al. classified macrophages as M1 (classically activated macrophages) and the (alternatively activated macrophages) based on their functions [1]. However, it is now known that the M1 and M2 nomenclature is too simplistic to describe the many distinct polarization phenotypes that are seen in tissue macrophages and are driven by many different environmental stimuli including cytokines, fatty acids, prostaglandins, and pathogen-derived molecules such as lipopolysaccharides (LPSs). Therefore, it is necessary to redefine this classification. In this review, we focused on the functions of macrophages in several pathological conditions from the point of view of phagocytosis.

## 2. Macrophages and Immunity

Various microorganisms inhabit the human body. Despite constant exposure to a wide range of microorganisms, the host immune system prevents the invasion of microbes. The human immune system consists of the innate and adaptive immune systems. Myeloid cells such as neutrophils, macrophages, and dendritic cells play a key role in the innate immune system by recognizing and removing bacteria. Furthermore, antigen-specific T cells and B cells function in the adaptive immune system to remove pathogens by cytotoxic reaction or producing antigen-specific antibodies. The innate immune system acts rapidly as the first line of defense. However, when this system is unable to destroy the pathogens completely, the adaptive immune system is activated.

The innate immune system destroys and excludes pathogens during early stages of the infection. Innate immunity is native to humans and indispensable for the maintenance of life. Innate immunity senses pathogens and stresses invading the body and removes them through an inflammatory response. Inflammatory reactions start when receptors belonging to innate immune cells recognize specific molecular patterns derived from pathogens or stresses. Pathogen-associated molecular patterns (PAMPs) are derived from microorganisms and are recognized by pattern recognition receptors (PRRs), which are found on innate immune cells as well as many epithelial cells. Conversely, damage-associated molecular patterns (DAMPs) initiate and activate an immune reaction in response to trauma, ischemia, or tissue damage regardless of the presence of a pathogenic infection [2]. Toll-like receptors (TLRs) are germline-encoded PRRs that play a central role in host cell recognition and responses to microbial pathogens. 

The discovery of the TLR family in the *Drosophila* fruit fly opened a path towards the elucidation of recognition mechanisms against microbial components by innate immunity cell [3]. TLRs recognize various kinds of components derived from bacteria or viruses as ligands. LPS, which is a component of the cell wall of Gram-negative bacteria, is recognized by the TLR4-MD2 complex, and bacterial lipopeptides and peptide glycans are recognized by heterodimeric TLR2/TLR1 and TLR2/TLR6. In addition, nucleic acids such as bacterial genomic DNA or viral RNA can be recognized by TLR3, TLR7, TLR8, and TLR9, which develop in the phagosome. The signals recognized by TLRs activate a downstream signal cascade through by adapter molecules such as myeloid differentiation primary response 88 (MyD88) and Toll/interleukin-1 receptor (TIR)-domain-containing adapter-inducing interferon-β (TRIF), and activate transcription factors such as NF-κB, resulting in the induction of gene expression [4]. 

In addition to the PRRs found on the cell membrane, different types of PRRs exist in the cytoplasm, including NOD-like receptors (NLRs), the HIN-200 receptor family, and RIG-1-like receptors (RLRs). Molecules functioning as stress sensors against cytoplasmic pathogens are included in these. Several NLRs, such as NALP1 and NALP3, form the inflammasome along with ASC and caspase-1 and mediate the processing of pro-interleukin (IL)-1β to mature IL-1β for release. It is thought that caspase-1 plays a role in the exclusion of infectious substances and the exacerbation of inflammation by activating pyroptosis, a highly inflammatory form of programmed cell death [5]. RLRs recognize viral double-stranded RNA and activate a downstream signal cascade through by interferon-β promoter stimulator (IPS-1). As a result, type I interferons and inflammatory cytokines are produced, which induce antiviral immune reaction [6]. As noted above, PRRs differ from each other in the mechanism of recognizing and subsequent immune response against various pathogens.

Recent studies have shown that the inflammasome forms a multiprotein complex of several hundred kDa in the cytoplasm. The complex is secreted outside the cell, and polymerized by inflammasome-related molecules. As a result, larger complexation is stimulated outside the cell. It was revealed that extensive complexation aggravated inflammation by activating the production of inflammatory cytokines [7,8]. Furthermore, activated macrophages phagocytose the inflammasome complex to further induce inflammation.

## 3. Mechanisms of Phagocytosis

**Figure 1:**
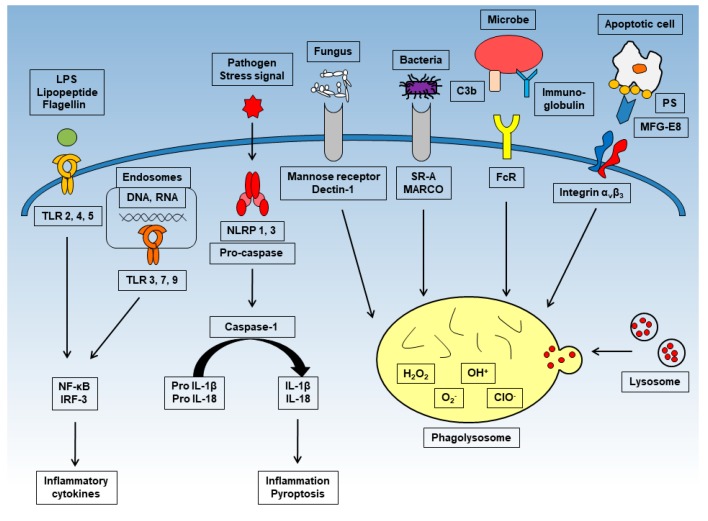
The inflammatory and phagocytic mechanisms of macrophages.

Phagocytes such as neutrophils, macrophages, and dendritic cells make a bridge between specific bacterial surface antigens and cellular receptors. Following this bridge, membrane protrusions surround the bacteria and absorb the bacteria into the phagosome, which is formed by the fusion of cell membranes [9]. Since there is a wide range of phagocytic receptors, a variety of signaling cascades can be activated during this process (Figure 1). These receptors have various degrees of ligand specificity, and can be classified based on the type of ligands they recognize: foreign molecules identifiable by unique molecular patterns, opsonins, and apoptotic bodies. Phagocytes have several PRRs that bind specifically to certain PAMPs. For instance, the mannose receptor and Dectin-1 induce the phagocytosis of fungi with particular polysaccharides on their surface [10,11]. In addition, several scavenger receptors initiate phagocytosis upon PAMP recognition; these include the scavenger receptor A (SR-A) and the macrophage receptor with collagenous structure (MARCO), which bind to the surface molecules of Gram-negative and -positive bacteria [12,13,14]. Several soluble molecules, called opsonins, can be deposited onto foreign surfaces and serve as adaptors that bind and activate potent phagocytic receptors. For instance, immunoglobulin G (IgG), when specifically bound to microbial surface antigens, associates with fragment crystallizable γ receptors (FcγRs) in phagocytes, which recognizes their fragment crystallizable (Fc) region [15,16]. The C3b and iC3b molecules of the complement system can also bind to foreign particles. In addition to the clearance of foreign particles, phagocytosis is important for cell turnover within the organism, as billions of cells die by apoptosis every day, which must be removed. The mechanism of phagocytosis is different depending whether the cells are apoptotic or non-apoptotic. It is thought that the best-characterized signature of apoptotic cells is an increased surface exposure of the lipid phosphatidylserine (PS) [17]. In non-apoptotic cells, PS is mostly restricted to the inner leaflet of the plasma membrane. However, once the apoptosis pathway is triggered, the concentration of PS on the outside leaflet of the plasma membrane increases 300-fold [18]. In addition, soluble proteins, such as milk-fat globular protein (MFG)-E8, growth arrest-specific protein (Gas)6, and protein S, bind to exposed PS and act as linkers, similar to opsonins [19,20].

After being absorbed into the phagosome through one of several methods of receptor stimulation, microbes and apoptotic cells are immediately exposed to oxygen-dependent and -independent attacks. Soon after absorption into the phagosome, NADPH-oxidase produces reactive oxygen species (ROS), and phospholipase A2 produces free fatty acids. These molecules show a sterilization effect against bacteria to some extent [21,22]. Thereafter, myeloperoxidase (MPO), a basic sterilization protein, various hydrolases, and lysosomes such as azurophilic granules fuse into the phagosome and degrade microbial or apoptotic cells. Next, the proton ATPase pump lowers the pH of the phagosome, activating a strong oxygen-dependent sterilization system, mainly through halogenation [23], or an oxygen-independent sterilization system with basic sterilization proteins and lysosomal enzymes [24,25]. Unusually, macrophages do not contain MPO, but instead have Fe^2+^ ions which bind to chelators such as adenosine as substitutes for MPO.

Macrophages and dendritic cells function as antigen-presenting cells (APCs). They present peptide antigens derived from digested bacteria on the major histocompatibility complex class II and activate acquired immunity by activating helper T cells. While macrophages present antigens within tissues, dendritic cells present antigens in the lymph node. Only dendritic cells can activate naïve T cells to become effector T cells, and are the most powerful APCs [26].

Degradation of pathogen releases additional ligands that can be detected by receptors on phagolysosomal membrane or in cytosol. For example, releasing bacterial DNA into cytosol activates TLR9 and increases inflammatory response. In addition, degradation of *S. aureus* peptidoglycan in phagosomes promotes activation of the NLRP3 inflammasome, but its precise mechanism has not yet been elucidated. A recent study has shown that release of bacterial components into cytosol enabled macrophages to sense microbial viability and mediate effective protection of host [27].

Toll-like receptor (TLR)-2, -4, and -5 are expressed on the cell surface and recognize bacterial components. TLR-3, -7, and -9 expressed in the endosome and recognize viral DNA and RNA. After stimulation, each induce inflammatory cytokines such as interleukin (IL)-6 or tumor necrosis factor (TNF)-α through NF-κB or interferon regulatory transcription factor (IRF)-3. NOD-like receptors (NLRs) such as NLRP-1 and -3 produce and activate caspase-1 and mediate the processing of pro-IL-1β to mature IL-1β. Mature IL-1β causes inflammation and pyroptosis. Phagocytosis is activated upon recognition of numerous antigens by several receptors. Representative receptors are shown in the figure. Through phagocytosis, harmful cells with antigens are digested and sterilized. 

## 4. Origins of Macrophages

Macrophages can be found in almost all organs in the body, including the liver, brain, bones, and lungs; they have specific functions in each organ. For instance, alveolar macrophages are necessary for processing surfactants, and macrophages in the gastrointestinal tract or adipose tissue play an important role in the maintenance of homeostasis. Thus, each organ and the surrounding environment influence their properties during differentiation. Until recently, it was believed that macrophages were derived from hematopoietic stem cells and differentiated into tissue-specific macrophages in local tissues. However, Takahashi et al. demonstrated that macrophages derived from embryonic yolk sacs were maintained in peripheral tissues by self-renewal, in addition to the macrophages derived from hematopoietic stem cells [28,29].

In 1968, van Furth et al. proposed that tissue-resident macrophages are continuously repopulated by blood-circulating monocytes, which arise from progenitors in adult bone marrow [30]. In the 2000s, several techniques were developed to identify macrophage subsets, including flow cytometry, DNA microarray analysis, and lineage analysis using genetically modified mice. These technical developments revealed several facts: (1) almost all microglia in the central nervous system are derived from the embryonic yolk sac and are maintained throughout life; (2) in the intestinal tract, macrophages derived from the embryonic yolk sac are replaced with macrophages from bone marrow monocytes immediately after birth; and (3) macrophages derived from the fetal liver are dominant in most tissues except the central nervous system and intestinal tract [26]. 

Macrophages activated by the invasion of pathogens to destroy them are categorized as M1 macrophages [31], and macrophages causing chronic inflammation because of allergic reactions, fat metabolism, wound healing, and cancer invasion and metastasis are categorized as M2 macrophages [32]. Generally, PAMPs, DAMPs, and inflammatory cytokines such as TNF-α and IFN-γ induce the M1 phenotype. Conversely, anti-inflammatory cytokines such as IL-10, IL-4, and IL-13 induce the M2 phenotype. Once the inflammatory reaction occurs, bone marrow monocytes infiltrate the inflammatory tissue and differentiate into macrophages or monocyte-derived dendritic cells. Generally, it is considered that embryonic-derived macrophages play a strong role in the maintenance of tissue homeostasis and that macrophages derived from bone marrow monocytes are related to host defense reactions and inflammatory diseases. Table 1 shows the origin of each tissue macrophages and cell surface marker.

Here, we describe the nature and function of tissue macrophages in different organs as well as tumors.

### 4.1. Lung

Humans breathe in air for gas exchange, but toxic substances such as bacteria, viruses, fungi, organic substances, and inorganic substances may be taken into the body through breathing. Macrophages and neutrophils process bacteria in the lung’s innate immunity. In a recent study, it was found that macrophages derived from the yolk sac reside in the peripheral interstitial tissue of lungs, macrophages derived from the fetal liver reside in the alveolae, and macrophages derived from bone marrow reside in the central interstitial tissue [33]. The alveolar macrophages contain the C lectin receptor group (mannose receptor, β-D glucan receptor, scavenger receptor, and complement receptor) at the cell surface, and these molecular groups recognize the invasion of pathogens. With respect to tuberculosis, TLR2 united with TLR1 or TLR6 is important in recognition. However, some tuberculosis is resistant to sterilization after phagocytosis by macrophages. In that case, macrophages and lymphocytes confine tuberculosis by the formation of granuloma. In the lung tissue, various factors such as complement, SP-A, SP-D, IgA, lysozymes, interferons, lactoferrins, defensins, LPS binding proteins, soluble CD14, chemokines, and cytokines work together to form the humoral immune system [34]. 

The production and maturation of alveolar macrophages depend on the transcription factor peroxisome proliferator-activated receptor gamma (PPAR-γ). The activation of PPAR-γ in alveolar macrophages depends on the activation of granulocyte-macrophage colony-stimulating factor (GM-CSF) in the lung. PPAR-γ not only acts as a transcription factor for the metabolism of lipids and carbohydrates like other PPARs but also plays an important role in inflammation, tissue repair, the degradation of surfactants, phagocytosis, and cell survival. PPAR-γ also induces the expression of the scavenger receptor CD36, which phagocytoses lipids and apoptotic neutrophils. In patients with idiopathic pulmonary alveolar proteinosis, in whom the expression of PPAR-γ is downregulated, and in GM-CSF knockout mouse alveolar macrophages, the expression of CD36 is also downregulated [35]. PPAR-γ ligand stimulation reinforces the FcγR-dependent phagocytosis of alveolar macrophages [36]. In addition, transforming growth factor (TGF)-β is also essential for the differentiation and homeostasis of alveolar macrophages. TGF-β controls alveolar macrophages in an autocrine-related manner. TGF-β also regulates the expression of genes associated with alveolar macrophage differentiation and fate [37].

The phagocytic ability of alveolar macrophages is impaired by several pathological conditions. For example, chronic alcohol ingestion causes dysfunction of alveolar macrophages. Alcohol downregulates the expression of GM-CSF receptors on the cell surface of the alveolar macrophages and impairs their immune function [38]. In HIV infection, alveolar macrophages are infected with HIV and infected cells have impaired phagocytic function as well as abnormal oxidative burst and cytokine secretion [39]. Thus, phagocytic ability of alveolar macrophages can be impaired in a different way.

### 4.2. Liver

The liver is always exposed to antigens from the gastrointestinal tract, including food-related antigens and PAMPs, through the hepatic portal vein. Intestinal bacteria (over 1 × 10^12^ cells) habitually reside in the gastrointestinal tract, and homeostasis in the body is maintained through immunoregulation mechanisms, which suppress the immune response to foreign antigens or bacterial components in the intestine. Kupffer cells are a self-sustaining population of macrophages in the liver derived from fetal liver monocyte and are distinguished from the monocyte-derived macrophages that rapidly accumulate in injured livers. 

Kupffer cells are seeded alongside sinusoidal endothelial cells and are important scavengers that constantly clear gut­derived pathogens from the blood [40]. To maintain homeostasis, Kupffer cells play a role in maintaining functional iron [41,42] and bilirubin metabolism [43]. Kupffer cells express Fc receptors and distinct scavenger receptors, allowing them to remove damaged erythrocytes, hemoglobin–haptoglobin complexes, and erythrocyte­derived hemoglobin­containing vesicles from the blood [44,45]. They also control cholesterol metabolism by, for example, expressing cholesteryl ester transfer protein (CETP), which is important for transferring cholesterol from high density lipoprotein (HDL) to very low-density lipoprotein (VLDL) [46].

In liver injuries, Kupffer cells secrete anti-inflammatory cytokines such as IL-10, IL-4, and IL-13 [47,48,49]. Interestingly, both resident and infiltrating macrophages derived from blood monocyte appear to cooperate in tissue repair [50]. Their beneficial actions include attenuating neutrophil-driven inflammation through prostaglandin (PGE)-2 synthesis, the phagocytosis of dead cells, and restoring vessel structures using angiogenesis factors such as vascular endothelial growth factor (VEGF)-A [51]. Both alveolar macrophages and Kupffer cells derive from fetal liver monocyte, but their response to surrounding stimuli is quite different as mentioned above. This suggests that surrounding environment could affect their differentiation of tissue macrophages. It was recently reported that peritoneal cavity macrophages that express GATA-binding protein 6 (GATA6) invaded liver tissue in response to a sterile injury and contributed to tissue repair by processing necrotic cells [52]. Additionally, activated Kupffer cells are known to be the major source of macrophage inflammatory protein-2 (MIP-2) in liver injuries. MIP-2 is one of the CXC chemokines and is also known as CXCL2. MIP-2 plays a dual role in mediating liver inflammation and promoting liver regeneration [53].

### 4.3. Brain

Microglia are derived from yolk sac erythromyeloid progenitors during primitive hematopoiesis in early embryonic development and are maintained at approximately 100% of their initial concentration throughout life. Microglia, a type of glial cell, rapidly sense neuropathy caused by brain damage, neurodegenerative diseases, or cerebral ischemia, and are activated to perform various functions [54]. Microglia express various receptors; LPS, peptide glycans, or viral glycoproteins bound by TLRs to activate the microglia. In addition, microglia express receptors for advanced glycation end-products (RAGE); additionally, amyloid-β protein and various aggregated proteins induce the activation of microglia [55]. A recent study revealed that DAMPs also activate microglia through TLRs [56]. 

Activated microglia produce not only several factors related to neuroprotection, such as various anti-inflammatory cytokines and neurotropic factors, but also produce nerve injury factors such as inflammatory cytokines, nitric oxide (NO), ROS, excitatory amino acids, and ATP. In Alzheimer’s disease, amyloid-β activates microglia to produce such nerve injury factors [57]. IL-6, IL-2, IL-3, GM-CSF, and erythropoietin have a direct effect on neuroprotection [58], while cytokines such as IL-1, IL-4, IL-5, and TGF-β have an indirect neuroprotection effect by inducing the production of nerve growth factor (NGF) in the astrocyte [59].

When neurons are damaged, microglia are activated and migrate to the damaged lesion. This migration is induced by ATP or ADP released from the damaged cells. It has been revealed that P2Y12 receptor, P2X4 receptor, and adenosine A1 and A3 receptors, which are expressed in microglia, participate in activating migration [60,61,62]. Activated microglia present an amoeboid form. Amoeboid microglia can phagocytose dead cells and foreign materials. It is thought that this phenomenon contributes to the environmental maintenance of the brain and tissue repair by preventing the release of non-essential and adverse factors.

### 4.4. Intestinal Tract

As mentioned earlier, macrophages in the intestinal tract are replaced by macrophages derived from bone marrow monocytes immediately after birth. Macrophages work as innate immune cells through phagocytosis and sterilization of foreign substances such as bacteria, and play a central role in defending the host from infection. However, residual macrophages in intestinal mucosa can potentially reduce inflammation to a greater extent than those in other tissues. Intestinal macrophages maintain their bacterial phagocytic and sterilization abilities, but do not express TLRs that recognize bacteria, leaving them unresponsive to bacterial antigens [63]. In addition, intestinal macrophages constantly produce IL-10 and directly participate in immunosuppression [64]. However, intestinal macrophages control the maintenance of the differentiation, proliferation, and function of peripheral regulatory T cells (pTregs) through IL-10 production [64,65,66]. Intestinal macrophages and pTregs are the primary IL-10-producing cells in the intestinal mucosa. Therefore, these networks of immune cells are important for immune tolerance to habitual antigens in the intestinal mucosa. Further, macrophage-derived IL-10 induces the secretion of the pro-repair WNT1-inducible signaling protein 1 (WISP-1) in response to intestinal mucosal injury. WISP-1 induces epithelial cell proliferation and wound repair by activating epithelial pro-proliferative pathways [67].

CD169^+^ macrophages in the splenic marginal zone play important roles in removing apoptotic blood cells and immune tolerance [68]. It was recently reported that CD169^+^ macrophages can be found in the gastrointestinal tract [69]. Generally, intestinal CD169^−^ macrophages are distributed to almost all areas of the lamina propria, while CD169^+^ macrophages are observed in the muscularis mucosae. Following mucosal injury, CD169^+^ macrophages produce CCL8 and that recruit inflammatory monocytes. The selective depletion of CD169^+^ macrophages or the administration of neutralizing anti-CCL8 antibody ameliorated clinical features and pathological tissue damage in an experimental colitis model in mice [70]. Thus, CCL8 derived from CD169^+^ macrophages serve as an emergency alert for broken barrier defense.

### 4.5. Spleen

The spleen is representative of organs with several different kinds of macrophages derived from fetal liver monocyte. The spleen contains discrete anatomical compartments, the red and white pulp regions, separated by a marginal zone. Tissue macrophages in the spleen can be distinguished as (1) red pulp macrophages, (2) marginal zone metallophilic macrophages distributed inside the marginal zone, (3) marginal zone macrophages distributed outside the marginal zone, and (4) tingible body macrophages distributed in the lymph follicle of the white pulp region [71,72]. 

Red pulp macrophages clear damaged blood cells, recycle iron [73,74], and catabolize heme [75]. Red pulp macrophages express Spi-C, a PU.1-related transcription factor. It has been revealed that Spi-C specifically regulates the differentiation of red pulp macrophages [76]. 

The germinal center of the white spleen is where B lymphocytes mature and differentiate. Cells expressing B-cell receptors with a high affinity for antigens survive, and those with low-affinity receptors undergo apoptosis [77]. CD68^+^ tingible body macrophages can be found in the germinal center of the white spleen and phagocytose apoptotic B lymphocytes. Tingible body macrophages specifically express MFG-E8 in the spleen. MFG-E8 is secreted by macrophages and recognizes phosphatide- and phosphatidylserine-presenting apoptotic cells [19,78]. 

Two kinds of tissue macrophages exist in the marginal zone. Marginal zone macrophages located outside the marginal zone have a superior phagocytic ability and develop PRRs such as macrophage receptor with collagenous structure (MARCO), scavenger receptor-A (SR-A), and SIGN-related 1 (SIGNR1), and play an important role in host defense by binding to various pathogens [79]. Furthermore, marginal zone metallophilic macrophages located inside the marginal zone develop CD169 molecules and participate in the immune response against neutral polysaccharides from bacterial compounds [80]. In addition, it was reported that the two kinds of marginal zone macrophages play an important role in immune tolerance instruction regarding the phagocytosis of dead cells [68,81].

### 4.6. Adipose Tissue

A large proportion of macrophage infiltrations have been identified between enlarged fat cells of adipose tissues in obese patients [82] and these macrophages derive from bone marrow monocyte. Almost all infiltrating macrophages are M1 macrophages, and the production of inflammatory cytokines such as tumor necrosis factor (TNF)-α and IL-6 is aggravated [83]. Inflammatory cytokines produced by the M1 macrophages act on surrounding fat cells and attenuate their sensitivity to insulin [84]. As a result, the fat cells have a decreased ability for glucose uptake, increasing the risk of diabetes. Macrophages exist in the adipose tissue of healthy individuals, but the M2 subtype is present [83]. M2 macrophages in adipose tissue participate in maintaining normal blood sugar levels through the secretion of insulin-like growth factor (IGF)-1 [85,86].

Free fatty acids, which are found at higher concentrations in obese patients, act on macrophages and control the inflammatory response. Palmitic acid, a saturated fatty acid, binds to TLR2 or TLR4 on the macrophage and induces an inflammatory response [87,88,89]. In addition, MCP-1 (CCL2), a member of the chemokine family, is upregulated in enlarged fat cells, as it induces the migration and infiltration of macrophages to adipose tissue [90,91].

### 4.7. Tumor

Since Virchow et al. revealed the existence of the white blood cell that infiltrates tumors in 1863, the relationship between tumor formation or growth and macrophages has been studied extensively. Tumor-associated macrophages (TAMs) are the main type of tumor-infiltrating cells, and the relationship between the number of TAMs in a tumor and the prognosis has been investigated in great detail [92]. In tumor tissues, CSF-1 or IL-10 produced in tumor cells induce the differentiation of TAMs to M2 macrophages [93]. M2-TAMs promote the growth and expansion of the tumor via various mechanisms. In tumor tissues, a drastic enhancement of vascular density promotes the oxygenation of and nutrient supply to tumor cells. Vascularization inducers such as VEGF, epidermal growth factor (EGF), TNF-α, basic fibroblast growth factor (FGF), platelet-derived growth factor (PDGF), thymidine phosphorylase, CXCL8, and CCL2 are produced in TAMs [94,95], and there are many reports indicating an association between the density of M2-TAMs and blood vessel density [92]. In addition, M2-TAMs suppress T cell-related antitumor immunity by producing immunosuppressive substances such as PGE2, TGF-β, and IL-10 [95] and control the maturation of dendritic cells through CSF-1, IL-6, and IL-10. In addition, growth factors such as basic FGF, hepatocyte growth factor (HGF), EGF, PDGF, and TGF-β produced from M2-TAMs promote the growth of tumor cells [93]. Recently, it was revealed that M2-TAMs also affected the formation of a niche to maintain cancer stem cell survival through these growth factors [96]. 

In the past, many reports have identified a correlation between the infiltration density of TAMs and poor prognosis in many cancers. It was reported that the infiltration density of M2-TAMs was more strongly related than the infiltration density of TAMs to a poor prognosis for breast cancer, pancreatic cancer, endometrial cancer, malignant melanoma, glioma, and malignant lymphoma [92]. 

Conversely, the infiltration of TAMs to surrounding tumors showed a low correlation to a poor prognosis in inflammation-driven cancers such as hepatocellular carcinoma, and cervical cancer, and there are many reports showing that high infiltration of (non-M2) TAMs was an indicator of a good prognosis for colon cancer. Although it is one of several possible mechanisms, the differentiation of TAMs to an M2 phenotype seems difficult under continuous exposure to intestinal flora [97]. 

## 5. Conclusions

Macrophages, the major population of tissue-resident mononuclear phagocytes, play key roles in bacterial recognition and elimination as well as in polarization of innate and adaptive immunity. Macrophages sometimes play a role in anti-inflammatory responses, tissue repair, and homeostasis while they sometimes promote inflammation and tumor growth. As we mentioned in this review, various types of macrophages regulate a wide variety of immune responses. To clarify the phagocytic mechanism of macrophages associated with abnormal inflammation and cancer immunity will contribute to the elucidation of several human diseases. Finally, manipulating macrophages based on their mechanism opens a way to the development of new therapies.

## Figures and Tables

**Table 1:** Origin of tissue macrophages and cell surface marker.

Tissue	Derived Cell in Adult	Cell Surface Marker
Lung	Fetal liver monocyte	F4/80^low^, CD11b^low^, CD11c^high^, CD68^+^, Siglec F^+^, MARCO^+^, CD206^+^, Dectine-1^+^
Liver	Fetal liver monocyte	F4/80^high^, CD11b^low^, CD169^+^, CD68^+^
Brain	Yolk sac macrophage	F4/80^+^, CD11b^+^, CD45^low^
Intestinal tract	Bone marrow monocyte	CXCR1^high^, F4/80^+^, CD11b^+^, CD11c^+^, CD64^+^
Spleen	Fetal liver monocyte	(red pulp macrophage) F4/80^+^, CD206^+^, Dectin-2^+^
		(Tingible-body macrophages) CD68^+^
		(Marginal zone macrophage) CD68^+^, CD209^+^, MARCO^+^, Dectin-2^+^, Tim4^+^
		(Marginal zone metallopilic macrophage) CD68^+^, CD169^+^, MOMA-1
Adipose tissue	Bone marrow monocyte	F4/80^+^, CD45^+^

## References

[1] Mills C.D., Kincaid K., Alt J.M., Heilman M.J., Hill A.M. (2000). M-1/M-2 Macrophages and the Th1/Th2 Paradigm. J. Immunol..

[2] Schenten D., Medzhitov R. (2011). The control of adaptive immune responses by the innate immune system. Adv. Immunol..

[3] Lemaitre B., Nicolas E., Michaut L., Reichhart J.M., Hoffmann J.A. (1996). The dorsoventral regulatory gene cassette spätzle/Toll/cactus controls the potent antifungal response in *Drosophila* adults. Cell.

[4] Kawai T., Akira S. (2011). Toll-like receptors and their crosstalk with other innate receptors in infection and immunity. Immunity.

[5] Martinon F., Mayor A., Tschopp J. (2009). The inflammasomes: Guardians of the body. Annu. Rev. Immunol..

[6] Bruns A.M., Horvath C.M. (2014). Antiviral RNA recognition and assembly by RLR family innate immune sensors. Cytokine Growth Factor Rev..

[7] Baroja-Mazo A., Martín-Sánchez F., Gomez A.I., Martínez C.M., Amores-Iniesta J., Compan V., Barberà-Cremades M., Yagüe J., Ruiz-Ortiz E., Antón J. (2014). The NLRP3 inflammasome is released as a particulate danger signal that amplifies the inflammatory response. Nat. Immunol..

[8] Franklin B.S., Bossaller L., De Nardo D., Ratter J.M., Stutz A., Engels G., Brenker C., Nordhoff M., Mirandola S.R., Al-Amoudi A. (2014). The adaptor ASC has extracellular and ‘prionoid’ activities that propagate inflammation. Nat. Immunol..

[9] Ueno N., Wilson M.E. (2012). Receptor-mediated phagocytosis of Leishmania: Implications for intracellular survival. Trends Parasitol..

[10] Herre J., Marshall A.S., Caron E., Edwards A.D., Williams D.L., Schweighoffer E., Tybulewicz V., Reis e Sousa C., Gordon S., Brown G.D. (2004). Dectin-1 uses novel mechanisms for yeast phagocytosis in macrophages. Blood.

[11] Ezekowitz R.A., Sastry K., Bailly P., Warner A. (1990). Molecular characterization of the human macrophage mannose receptor: Demonstration of multiple carbohydrate recognition-like domains and phagocytosis of yeasts in Cos-1 cells. J. Exp. Med..

[12] Van der Laan L.J., Döpp E.A., Haworth R., Pikkarainen T., Kangas M., Elomaa O., Dijkstra C.D., Gordon S., Tryggvason K., Kraal G. (1999). Regulation and functional involvement of macrophage scavenger receptor MARCO in clearance of bacteria in vivo. J. Immunol..

[13] Peiser L., Gough P.J., Kodama T., Gordon S. (2000). Macrophage class A scavenger receptor-mediated phagocytosis of *Escherichia coli*: Role of cell heterogeneity, microbial strain, and culture conditions in vitro. Infect. Immun..

[14] Thomas C.A., Li Y., Kodama T., Suzuki H., Silverstein S.C., El Khoury J. (2000). Protection from lethal gram-positive infection by macrophage scavenger receptor-dependent phagocytosis. J. Exp. Med..

[15] Anderson C.L., Shen L., Eicher D.M., Wewers M.D., Gill J.K. (1990). Phagocytosis mediated by three distinct Fc γ receptor classes on human leukocytes. J. Exp. Med..

[16] Nimmerjahn F., Ravetch J.V. (2008). Fcγ receptors as regulators of immune responses. Nat. Rev. Immunol..

[17] Fadok V.A., Voelker D.R., Campbell P.A., Cohen J.J., Bratton D.L., Henson P.M. (1992). Exposure of phosphatidylserine on the surface of apoptotic lymphocytes triggers specific recognition and removal by macrophages. J. Immunol..

[18] Borisenko G.G., Matsura T., Liu S.X., Tyurin V.A., Jianfei J., Serinkan F.B., Kagan V.E. (2003). Macrophage recognition of externalized phosphatidylserine and phagocytosis of apoptotic Jurkat cells—Existence of a threshold. Arch. Biochem. Biophys..

[19] Hanayama R., Tanaka M., Miwa K., Shinohara A., Iwamatsu A., Nagata S. (2002). Identification of a factor that links apoptotic cells to phagocytes. Nature.

[20] Anderson H.A., Maylock C.A., Williams J.A., Paweletz C.P., Shu H., Shacter E. (2003). Serum-derived protein S binds to phosphatidylserine and stimulates the phagocytosis of apoptotic cells. Nat. Immunol..

[21] Roos D. (1991). The involvement of oxygen radicals in microbicidal mechanisms of leukocytes and macrophages. Klin. Wochenschr..

[22] Akaki T., Sato K., Shimizu T., Sano C., Kajitani H., Dekio S., Tomioka H. (1997). Effector molecules in expression of the antimicrobial activity of macrophages against Mycobacterium avium complex: Roles of reactive nitrogen intermediates, reactive oxygen intermediates, and free fatty acids. J. Leukoc. Biol..

[23] Klebanoff S.J. (1968). Myeloperoxidase-halide-hydrogen peroxide antibacterial system. J. Bacteriol..

[24] Spitznagel J.K. (1990). Antibiotic proteins of human neutrophils. J. Clin. Investig..

[25] Hiemstra P.S. (2006). Defensins and cathelicidins in inflammatory lung disease: Beyond antimicrobial activity. Biochem. Soc. Trans..

[26] Guilliams M., Ginhoux F., Jakubzick C., Naik S.H., Onai N., Schraml B.U., Segura E., Tussiwand R., Yona S. (2014). Dendritic cells, monocytes and macrophages: A unified nomenclature based on ontogeny. Nat. Rev. Immunol..

[27] Underhill D.M., Goodridge H.S. (2012). Information processing during phagocytosis. Nat. Rev. Immunol..

[28] Takahashi K., Yamamura F., Naito M. (1989). Differentiation, maturation, and proliferation of macrophages in the mouse yolk sac: A light-microscopic, enzyme-cytochemical, immunohistochemical, and ultrastructural study. J. Leukoc. Biol..

[29] Ginhoux F., Guilliams M. (2016). Tissue-Resident Macrophage Ontogeny and Homeostasis. Immunity.

[30] Van Furth R., Cohn Z.A., Hirsch J.G., Humphrey J.H., Spector W.G., Langevoort H.L. (1972). The mononuclear phagocyte system: A new classification of macrophages, monocytes, and their precursor cells. Bull. World Health Organ..

[31] Takeuchi O., Akira S. (2010). Pattern recognition receptors and inflammation. Cell.

[32] Gordon S., Martinez F.O. (2010). Alternative activation of macrophages: Mechanism and functions. Immunity.

[33] Tan S.Y., Krasnow M.A. (2016). Developmental origin of lung macrophage diversity. Development.

[34] Martin T.R., Frevert C.W. (2005). Innate immunity in the lungs. Proc. Am. Thorac. Soc..

[35] Thomassen M.J., Barna B.P., Malur A.G., Bonfield T.L., Farver C.F., Malur A., Dalrymple H., Kavuru M.S., Febbraio M. (2007). ABCG1 is deficient in alveolar macrophages of GM-CSF knockout mice and patients with pulmonary alveolar proteinosis. J. Lipid Res..

[36] Aronoff D.M., Serezani C.H., Carstens J.K., Marshall T., Gangireddy S.R., Peters-Golden M., Reddy R.C. (2007). Stimulatory Effects of Peroxisome Proliferator-Activated Receptor-γ on Fcγ Receptor-Mediated Phagocytosis by Alveolar Macrophages. PPAR Res..

[37] Yu X., Buttgereit A., Lelios I., Utz S.G., Cansever D., Becher B., Greter M. (2017). The Cytokine TGF-β Promotes the Development and Homeostasis of Alveolar Macrophages. Immunity.

[38] Mehta A.J., Guidot D.M. (2017). Alcohol and the Lung. Alcohol Res..

[39] Presti R.M., Flores S.C., Palmer B.E., Atkinson J.J., Lesko C.R., Lau B., Fontenot A.P., Roman J., McDyer J.F., Twigg H.L. (2017). Mechanisms Underlying HIV-Associated Noninfectious Lung Disease. Chest.

[40] Tacke F., Zimmermann H.W. (2014). Macrophage heterogeneity in liver injury and fibrosis. J. Hepatol..

[41] Gammella E., Buratti P., Cairo G., Recalcati S. (2014). Macrophages: Central regulators of iron balance. Metallomics.

[42] Theurl M., Theurl I., Hochegger K., Obrist P., Subramaniam N., van Rooijen N., Schuemann K., Weiss G. (2008). Kupffer cells modulate iron homeostasis in mice via regulation of hepcidin expression. J. Mol. Med..

[43] Naito M., Hasegawa G., Ebe Y., Yamamoto T. (2004). Differentiation and function of Kupffer cells. Med. Electron. Microsc..

[44] Willekens F.L., Were J.M., Kruijt J.K., Roerdinkholder-Stoelwinder B., Groenen-Döpp Y.A., van den Bos A.G., Bosman G.J., van Berkel T.J. (2005). Liver Kupffer cells rapidly removered blood cell-derived vesicles from the circulation by scavenger receptors. Blood.

[45] Kristiansen M., Graversen J.H., Jacobsen C., Sonne O., Hoffman H.J., Law S.K., Moestrup S.K. (2001). Identification of the haemoglobin scavenger receptor. Nature.

[46] Wang Y., van der Tuin S., Tjeerdema N., van Dam A.D., Rensen S.S., Hendrikx T., Berbée J.F., Atanasovska B., Fu J., Hoekstra M. (2015). Plasma cholesteryl ester transfer protein is predominantly derived from Kupffer cells. Hepatology.

[47] Ju C., Tacke F. (2016). Hepatic macrophages in homeostasis and liver diseases: From pathogenesis to novel therapeutic strategies. Cell. Mol. Immunol..

[48] Bourdi M., Masubuchi Y., Reilly T.P., Amouzadeh H.R., Martin J.L., George J.W., Shah A.G., Pohl L.R. (2002). Protection against Acetaminophen-Induced Liver Injury and Lethality by Interleukin 10: Role of Inducible Nitric Oxide Synthase. Hepatology.

[49] Sutter A.G., Palanisamy A.P., Ellet J.D., Schmidt M.G., Schnellmann R.G., Chavin K.D. (2014). Intereukin-10 and Kupffer cells protect steatotic mice livers from ischemia-reperfusion injury. Eur. Cytokine Netw..

[50] You Q., Holt M., Yin H., Li G., Hu C.J., Ju C. (2013). Role of hepatic resident and infiltrating macrophages in liver repair after acute injury. Biochem. Pharmacol..

[51] Krenkel O., Mossanen J.C., Tacke F. (2014). Immune mechanisms in acetaminophen-induced acute liver failure. Hepatobiliary Surg. Nutr..

[52] Wang J., Kubes P. (2016). A Reservoir of Mature Cavity Macrophages that Can Rapidly Invade Visceral Organs to Affect Tissue Repair. Cell.

[53] Qin C.C., Liu Y.N., Hu Y., Yang Y., Chen Z. (2017). Macrophage inflammatory protein-2 as mediator of inflammation in acute liver injury. World J. Gastroenterol..

[54] Kreutzberg G.W. (1996). Microglia: A sensor for pathological events in the CNS. Trends Neurosci..

[55] Yan S.S., Chen D., Yan S., Guo L., Du H., Chen J.X. (2012). RAGE is a key cellular target for Aβ-induced perturbation in Alzheimer’s disease. Front. Biosci..

[56] Kigerl K.A., de Rivero Vaccari J.P., Dietrich W.D., Popovich P.G., Keane R.W. (2014). Pattern recognition receptors and central nervous system repair. Exp. Neurol..

[57] Yu Y., Ye R.D. (2015). Microglial Aβ receptors in Alzheimer’s disease. Cell. Mol. Neurobiol..

[58] Gao W., Li F., Zhou Z., Xu X., Wu Y., Zhou S., Yin D., Sun D., Xiong J., Jiang R. (2017). IL-2/Anti-IL-2 Complex Attenuates Inflammation and BBB Disruption in Mice Subjected to Traumatic Brain Injury. Front. Neurol..

[59] Nakajima K., Tohyama Y., Maeda S., Kohsaka S., Kurihara T. (2007). Neuronal regulation by which microglia enhance the production of neurotrophic factors for GABAergic, catecholaminergic, and cholinergic neurons. Neurochem. Int..

[60] Ohsawa K., Irino Y., Nakamura Y., Akazawa C., Inoue K., Kohsaka S. (2007). Involvement of P2X4 and P2Y12 receptors in ATP-induced microglial chemotaxis. Glia.

[61] Ohsawa K., Sanagi T., Nakamura Y., Suzuki E., Inoue K., Kohsaka S. (2012). Adenosine A3 receptor is involved in ADP-induced microglial process extension and migration. J. Neurochem..

[62] Sasaki Y., Hoshi M., Akazawa C., Nakamura Y., Tsuzuki H., Inoue K., Kohsaka S. (2003). Selective expression of Gi/o-coupled ATP receptor P2Y12 in microglia in rat brain. Glia.

[63] Smythies L.E., Sellers M., Clements R.H., Mosteller-Barnum M., Meng G., Benjamin W.H., Orenstein J.M., Smith P.D. (2005). Human intestinal macrophages display profound inflammatory anergy despite avid phagocytic and bacteriocidal activity. J. Clin. Investig..

[64] Denning T.L., Wang Y.C., Patel S.R., Williams I.R., Pulendran B. (2007). Lamina propria macrophages and dendritic cells differentially induce regulatory and interleukin 17-producing T cell responses. Nat. Immunol..

[65] Murai M., Turovskaya O., Kim G., Madan R., Karp C.L., Cheroutre H., Kronenberg M. (2009). Interleukin 10 acts on regulatory T cells to maintain expression of the transcription factor Foxp3 and suppressive function in mice with colitis. Nat. Immunol..

[66] Shouval D.S., Biswas A., Goettel J.A., McCann K., Conaway E., Redhu N.S., Mascanfroni I.D., Al Adham Z., Lavoie S., Ibourk M. (2014). Interleukin-10 receptor signaling in innate immune cells regulates mucosal immune tolerance and anti-inflammatory macrophage function. Immunity.

[67] Quiros M., Nishio H., Neumann P.A., Siuda D., Brazil J.C., Azcutia V., Hilgarth R., O’Leary M.N., Garcia-Hernandez V., Leoni G. (2017). Macrophage-derived IL-10 mediates mucosal repair by epithelial WISP-1 signaling. J. Clin. Investig..

[68] Miyake Y., Asano K., Kaise H., Uemura M., Nakayama M., Tanaka M. (2007). Critical role of macrophages in the marginal zone in the suppression of immune responses to apoptotic cell-associated antigens. J. Clin. Investig..

[69] Hiemstra I.H., Beijer M.R., Veninga H., Vrijland K., Borg E.G., Olivier B.J., Mebius R.E., Kraal G., den Haan J.M. (2014). The identification and developmental requirements of colonic CD169^+^ macrophages. Immunology.

[70] Asano K., Takahashi N., Ushiki M., Monya M., Aihara F., Kuboki E., Moriyama S., Iida M., Kitamura H., Qiu C.H. (2015). Intestinal CD169^+^ macrophages initiate mucosal inflammation by secreting CCL8 that recruits inflammatory monocytes. Nat. Commun..

[71] Gordon S., Taylor P.R. (2005). Monocyte and macrophage heterogeneity. Nat. Rev. Immunol..

[72] Mebius R.E., Kraal G. (2005). Structure and function of the spleen. Nat. Rev. Immunol..

[73] Soares M.P., Hamza I. (2016). Macrophages and Iron Metabolism. Immunity.

[74] Franken L., Klein M., Spasova M., Elsukova A., Wiedwald U., Welz M., Knolle P., Farle M., Limmer A., Kurts C. (2015). Splenic red pulp macrophages are intrinsically superparamagnetic and contaminate magnetic cell isolates. Sci. Rep..

[75] Haldar M., Kohyama M., So A.Y., Kc W., Wu X., Briseño C.G., Satpathy A.T., Kretzer N.M., Arase H., Rajasekaran N.S. (2014). Heme-mediated SPI-C induction promotes monocyte differentiation into iron-recycling macrophages. Cell.

[76] Kohyama M., Ise W., Edelson B.T., Wilker P.R., Hildner K., Mejia C., Frazier W.A., Murphy T.L., Murphy K.M. (2009). Role for Spi-C in the development of red pulp macrophages and splenic iron homeostasis. Nature.

[77] Victora G.D., Nussenzweig M.C. (2012). Germinal centers. Annu. Rev. Immunol..

[78] Hanayama R., Tanaka M., Miyasaka K., Aozasa K., Koike M., Uchiyama Y., Nagata S. (2004). Autoimmune disease and impaired uptake of apoptotic cells in MFG-E8-deficient mice. Science.

[79] Taylor P.R., Martinez-Pomares L., Stacey M., Lin H.H., Brown G.D., Gordon S. (2005). Macrophage receptors and immune recognition. Annu. Rev. Immunol..

[80] Taylor P.R., Gordon S., Martinez-Pomares L. (2005). The mannose receptor: Linking homeostasis and immunity through sugar recognition. Trends Immunol..

[81] McGaha T.L., Karlsson M.C. (2016). Apoptotic cell responses in the splenic marginal zone: A paradigm for immunologic reactions to apoptotic antigens with implications for autoimmunity. Immunol. Rev..

[82] Weisberg S.P., McCann D., Desai M., Rosenbaum M., Leibel R.L., Ferrante A.W. (2003). Obesity is associated with macrophage accumulation in adipose tissue. J. Clin. Investig..

[83] Lumeng C.N., Bodzin J.L., Saltiel A.R. (2007). Obesity induces a phenotypic switch in adipose tissue macrophage polarization. J. Clin. Investig..

[84] Tilg H., Moschen A.R. (2008). Inflammatory mechanisms in the regulation of insulin resistance. Mol. Med..

[85] Satoh T., Kidoya H., Naito H., Yamamoto M., Takemura N., Nakagawa K., Yoshioka Y., Morii E., Takakura N., Takeuchi O. (2013). Critical role of Trib1 in differentiation of tissue-resident M2-like macrophages. Nature.

[86] Spadaro O., Camell C.D., Bosurgi L., Nguyen K.Y., Youm Y.H., Rothlin C.V., Dixit V.D. (2017). IGF1 Shapes Macrophage Activation in Response to Immunometabolic Challenge. Cell Rep..

[87] Nguyen M.T., Favelyukis S., Nguyen A.K., Reichart D., Scott P.A., Jenn A., Liu-Bryan R., Glass C.K., Neels J.G., Olefsky J.M. (2007). A subpopulation of macrophages infiltrates hypertrophic adipose tissue and is activated by free fatty acids via Toll-like receptors 2 and 4 and JNK-dependent pathways. J. Biol. Chem..

[88] Lee J.Y., Zhao L., Youn H.S., Weatherill A.R., Tapping R., Feng L., Lee W.H., Fitzgerald K.A., Hwang D.H. (2004). Saturated fatty acid activates but polyunsaturated fatty acid inhibits Toll-like receptor 2 dimerized with Toll-like receptor 6 or 1. J. Biol. Chem..

[89] Shi H., Kokoeva M.V., Inouye K., Tzameli I., Yin H., Flier J.S. (2006). TLR4 links innate immunity and fatty acid-induced insulin resistance. J. Clin. Investig..

[90] Lazar M.A. (2006). The humoral side of insulin resistance. Nat. Med..

[91] Sárvári A.K., Doan-Xuan Q.M., Bacsó Z., Csomós I., Balajthy Z., Fésüs L. (2015). Interaction of differentiated human adipocytes with macrophages leads to trogocytosis and selective IL-6 secretion. Cell Death Dis..

[92] Takeya M., Komohara Y. (2016). Role of tumor-associated macrophages in human malignancies: Friend or foe?. Pathol. Int..

[93] Pollard J.W. (2004). Tumour-educated macrophages promote tumour progression and metastasis. Nat. Rev. Cancer.

[94] Quail D.F., Joyce J.A. (2013). Microenvironmental regulation of tumor progression and metastasis. Nat. Med..

[95] Allavena P., Sica A., Garlanda C., Mantovani A. (2008). The Yin-Yang of tumor-associated macrophages in neoplastic progression and immune surveillance. Immunol. Rev..

[96] Raggi C., Mousa H.S., Correnti M., Sica A., Invernizzi P. (2016). Cancer stem cells and tumor-associated macrophages: A roadmap for multitargeting strategies. Oncogene.

[97] Edin S., Wikberg M.L., Oldenborg P.A., Palmqvist R. (2013). Macrophages: Good guys in colorectal cancer. Oncoimmunology.

